# Effects of Hydroalcoholic Extract of Soy on Learning, Memory and Synaptic Plasticity Deficits Induced by Seizure in Ovariectomized Rats

**DOI:** 10.18869/nirp.bcn.8.5.395

**Published:** 2017

**Authors:** Mehdi Khodamoradi, Majid Asadi-Shekaari, Saeed Esmaeili-Mahani, Fariba Sharififar, Vahid Sheibani

**Affiliations:** 1. Neuroscience Research Center, Institute of Neuropharmacology, Kerman University of Medical Sciences, Kerman, Iran.; 2. Department of Biology, Faculty of Science, Shahid Bahonar University, Kerman, Iran.; 3. Herbal and Traditional Medicines Research Center, Faculty of Pharmacy, Kerman University of Medical Sciences, Kerman, Iran.

**Keywords:** Seizure, Soy extract, Genistein, Long-term potentiation, Spatial memory

## Abstract

**Introduction:**

Previous studies have shown that seizure can induce cognitive impairment. On the other hand, soy phytoestrogens, which are mainly found in soybean (Glycine max (L.) Merr.), have beneficial effects on the nervous system. However, little is known about their probable effects on seizure. The present study aimed to examine the probable effects of soy extract, containing the phytoestrogen genistein on seizure-induced cognitive and synaptic plasticity impairment in Ovariectomized (OVX) rats.

**Methods:**

Rats were ovariectomized, implanted with guide cannula and then divided into 5 groups (n=7–8 in each group): PBS, KA, Saline-KA, Higher Dose Soy (HDS-KA), and Lower Dose Soy (LDS-KA) groups. Animals of the HDS-KA and LDS-KA groups received intraperitoneal administration of soy extract (20 and 2 mg/kg, respectively) and the Saline-KA group received normal saline once a day for 4 days. Sixty minutes after the last injection, Kainic Acid (KA) or PBS was injected into the left lateral ventricle via pre-implanted guide cannula to induce generalized seizures. The Morris water maze task and in vivo field potential recordings were conducted 7 days later.

**Results:**

Soy extract at both doses significantly improved learning impairment and at the higher dose (20 mg/kg) significantly prevented seizure-induced spatial memory impairment and deficit of long-term potentiation in the hippocampus.

**Conclusion:**

The soy extract containing the phytoestrogen genistein may have beneficial effects on memory deficit induced by seizure in OVX rats and this effect is accompanied by a beneficial effect on synaptic plasticity.

## 1. Introduction

Seizure is a temporal and abnormal neuronal hyperactivity in the brain which may lead to neuronal damage, synaptic rearrangement and memory impairment (Artinian, Peret, Mircheva, Marti, & Crepel, 2015; Ben-Ari, 2001; Chan et al., 2004; Nadler, Perry, & Cotman, 1978; Vasiliev et al., 2014). In the brain, the hippocampus has a crucial role in memory formation; therefore, hippocampal damage following seizure can result in memory deficit (Kotloski, Lynch, Lauersdorf, & Sutula, 2002; Neves, Cooke, & Bliss, 2008). Furthermore, inflammation, free radicals production induced by oxidative stress, and neuronal loss following seizure in the hippocampus can result in memory deficit (Ben-Ari, 2001; Chan et al., 2004; Patel & Li, 2003; Shapiro, Wang, & Ribak, 2008). Long-Term Potentiation (LTP) in the hippocampus is accepted as a synaptic model for memory, which is also impaired by seizure (Bliss & Collingridge, 1993; Palizvan, Fathollahi, Semnanian, Hajezadeh, & Mirnajafizadeh, 2001; Zhang et al., 2010).

Soybean (Glycine max (L.) Merr.) consumption is common in Southeast Asia since a few thousand years ago because of its beneficial effects on health. Such effects of soybean may be due to its ingredients like oil, proteins, essential amino acids and secondary metabolites such as phenolic compounds and flavonoids. The phytoestrogens are the principle compounds of the isoflavones group, which have beneficial properties, like protecting neurons; improving cognitive function; prevention of cancer, cardiovascular diseases, osteoporosis, and menopause symptoms. Phytoestrogens, especially genistein, which are mainly found in soybeans, have similar structure and function to that of estrogen (Bolanho & Beléia, 2011; Barnes, 1998; Duffy, Wiseman, & File, 2003; Molteni, Brizio-Molteni, & Persky, 1995; Setchell & Cole, 2003). Therefore, some women tend to use Soy Phytoestrogens (SPEs) after menopause because of their beneficial effects against reduction of ovarian hormones (Hammond, 1994; Molteni et al., 1995; Soni et al., 2014). Ovariectomized (OVX) rodents are commonly used to provide the experimental conditions of menopause (Gallo et al., 2005; Kalu, 1991).

Soy extract containing phytoestrogens improves cognitive performance in postmenopausal women (Duffy et al., 2003). Also, soy extract and genistein attenuate hippocampal neurodegeneration following Kainic Acid (KA) administration in OVX rats (Azcoitia, Moreno, Carrero, Palacios, & Garcia-Segura, 2006). Nevertheless, there are a few studies about the effects of SPEs on seizure-induced memory deficit. This study aimed to assess the impact of soy extract containing the phytoestrogen genistein on memory and hippocampal LTP deficits following kainate-induced seizure in OVX rats.

## 2. Methods

### 2.1. Subjects

In this investigation, 68 female Wistar rats, aged 3–4 months, were obtained from the animal house of the Kerman Neuroscience Research Center. They were provided with water and food at libitum and maintained at 23°C±1°C, under 12:12 h light-dark cycle. All experiments were confirmed by the Regional Ethics Committee of Kerman Neuroscience Research Center (EC/KNRC/91-36) according to the “NIH Guide for the Care and Use of Laboratory Animals.”

### 2.2. Extract preparation

Soy plant was collected from Golestan Province (northern Iran) and authenticated by botanists of the Pharmacognosy Department of the Kerman University of Medical Sciences, where a voucher was deposited (Herbarium No. KF1521). The hydroalcoholic extract was prepared as per described in a previous study (Ebrahimzadeh Bideskan et al., 2011). To assess the genistein content of the extract, 1 mg of genistein was sonicated by 10 mL methanol for 5 minutes. A volume of 2 mL of the stock solution was added to 2 mL of AlCl_3_ 2% (methanolic solution, w/v). They were incubated for 30 minutes and then absorption spectra of genistein was determined and ƛmax 270 nm of the absorption was provided using an UV-Visible spectrophotometer (Lambda 25, Perkin Elmer, USA). The absorbance versus concentration curve was plotted using different concentrations of the stock solution (1.25, 2.5, 5, 7.5, 10 ppm). A stock solution of the extract (at 100 ppm) was added to an equal volume of AlCl_3_ 2% and after 30 minutes of incubation the absorbance was measured. This solution was used to determine the genistein content of the extract.

### 2.3. Ovariectomy

After intraperitoneal administration of ketamine (60 mg/kg) and xylazine (10 mg/kg), sufficient anesthesia was confirmed by response to gentle pinching of foot pad and reduction of respiratory rate. Afterwards, laparotomy was done and the ovaries were removed. The OVX animals were returned to their cages for recovery (Ebrahimzadeh Bideskan et al., 2011; Saadati et al., 2015).

### 2.4. Surgery and treatment procedure

Three weeks later, the animals were surgically implanted with unilateral guide cannulae (21-gauge, stainless steel) targeting at 1 mm over the lateral ventricle of the left hemisphere (AP=0.9 mm; ML=1.3 mm; DV=3.5 mm) (Paxinos & Watson, 2006). Seven days later, the animals were divided into 5 groups by random: PBS, KA, Saline-KA, Higher Dose Soy (HDS-KA), and Lower Dose Soy (LDS-KA) groups. Each group was then divided into two subgroups for the Morris Water Maze (MWM) task (n=7–8) and field potential recordings (n=7–8). Before intracerebroventricular (i.c.v.) infusion of kainate, the animals were injected intraperitoneally by soy extract at two doses (2 mg/kg for the LDS-KA groups and 20 mg/kg for the HDS-KA groups) (Azcoitia et al., 2006) or by normal saline (in the Saline-KA group) once a day for four consecutive days. In the KA groups, the animals only received kainate and in the PBS groups, the animals received 0.1 M Phosphate Buffered Saline (PBS). KA (K-0250) (0.5 μg/1μL) (Li et al., 2010; Nadler et al., 1978) was infused slowly into the left lateral ventricle one hour after the last administration of soy extract or normal saline.

The i.c.v. infusion was done via a microinjection cannula and a 10-μL Hamilton syringe. Seizure stages were classified as follows: 0- no behavioral change, 1- myoclonic jerks, 2- minimal seizures, 3- forelimb clonus, 4- rearing, and 5- rearing followed by falling (Racine, 1972). Seizure activity was observed for 3–4 h after KA administration and only that animals which reached the fifth stage were considered for the MWM task and field potential recordings which were performed one week later. It is stated that i.c.v. infusion of kainate induces severe hippocampal damage after 7 days (Li et al., 2010).

### 2.5. MWM

#### 2.5.1. Acquisition

A black pool (diameter=160 cm, and height=80 cm) was filled with water to a height of 40 cm in a dimly lit room. The temperature of water was kept at 22°C±1°C during the experiments. Four quadrants were considered for the pool and a platform (10 cm in diameter) submerged below the water surface (∼1.5 cm) in the target quadrant. Spatial navigation cues were attached on the walls around the maze. For spatial learning, three blocks (each of the blocks included four trials, each 60 s in duration, with intertrial intervals, 60 s in duration) were conducted with a 30-min resting period between the blocks. In all trials, the rats were released into the water from one of the quadrants. If a rat failed to find the hidden platform within 60 s, they were guided by experimenter to find it. The path length and escape latency to discover the platform were examined to assess spatial learning. The animals’ performance was measured using a video tracking system (Noldus Ethovision® system, version 5, USA) (Ghazvini et al., 2016; Hajali et al., 2015; Saadati et al., 2015).

#### 2.5.2. Retention

Two hours after the last acquisition trial, the probe test (one trial with removed platform) was performed to assess spatial memory (the traveled distance and amount of time taken in the target quadrant). Afterwards, the visible platform test was conducted to examine whether or not seizure induction has interfered with motivation or sensory and motor coordination. The platform was covered with an aluminum foil and placed ∼2 cm over the water surface and the amount of time taken to discover the visible platform was measured (Ghazvini et al., 2016; Hajali et al., 2015; Saadati et al., 2015).

### 2.6. Field potential recording

Field potentials were recorded as described previously (Hajali et al., 2015; Saadati, Sheibani, Esmaeili-Mahani, Hajali, & Mazhari, 2014). After anesthesia with urethane (1.2 g/kg), the animals were surgically implanted with stimulating and recording electrodes in the right hippocampus (Astur, Taylor, Mamelak, Philpott, & Sutherland, 2002; Saadati et al., 2014). An electrode (0.125 mm diameter, Advent, UK) was placed in the Schaffer collateral pathway (AP=3 mm; ML=3.5 mm; DV=2.8–3 mm) for stimulation and the other electrode was placed in the stratum radiatum of CA1 area of the same side (AP=4.1; ML=3 mm; DV=2.5 mm) (Paxinos & Watson, 2006) for recording. The electrode used for stimulation was coupled to a stimulator and the electrode used for recording was coupled to an amplifier.

The stimulus intensity was gradually increased and the Field Excitatory Postsynaptic Potentials (fEPSPs) were recorded to provide the input-output curve. The fEPSPs were amplified and filtered (1 to 3000 Hz band pass filters). Using Neurotrace software (version 9), the baseline was recorded for 20 min by delivering a test stimulus every 10 s. Then, paired-pulse relationships were evaluated at 20, 50, 70 and 100 ms inter-stimulus intervals (averaged from ten sequential paired-pulses) and fEPSP slope ratios were then evaluated (fEPSP2/fEPSP1). Afterwards, high frequency stimulation (HFS, 10 pulses at 400 Hz/7 s repeated for 70 s) was used to induce LTP. The maintenance phase was then recorded by applying a test stimulus every 10 s for 120 min (Hajali et al., 2015; Saadati et al., 2014). The data were analyzed using Potentalize software.

### 2.7. Statistical analysis

The time and distance to discover the hidden platform were analyzed using 2-way analysis of ANOVA and repeated measurements (groups and blocks as the factors). One-way ANOVA and Tukey test were used to assess the time spent, traveled distance, the number of crossings in the target quadrant, and swimming speed. The repeated measure ANOVA was used to analyze the overall differences (at all time points) among the groups (group and time as the factor) and 1-way ANOVA and Tukey test were used to analyze the single time points (5 and 120 min after HFS) among the groups. The obtained data are expressed as mean and Standard Error of the Mean (S.E.M.) and P less than 0.05 were considered statistically significant.

## 3. Results

### 3.1. Genistein content of the extract

The genistein content of the soy extract was estimated to be 1.22% (g/g).

### 3.2. Behavioral study

#### 3.2.1. Spatial learning

**Figure 1.**
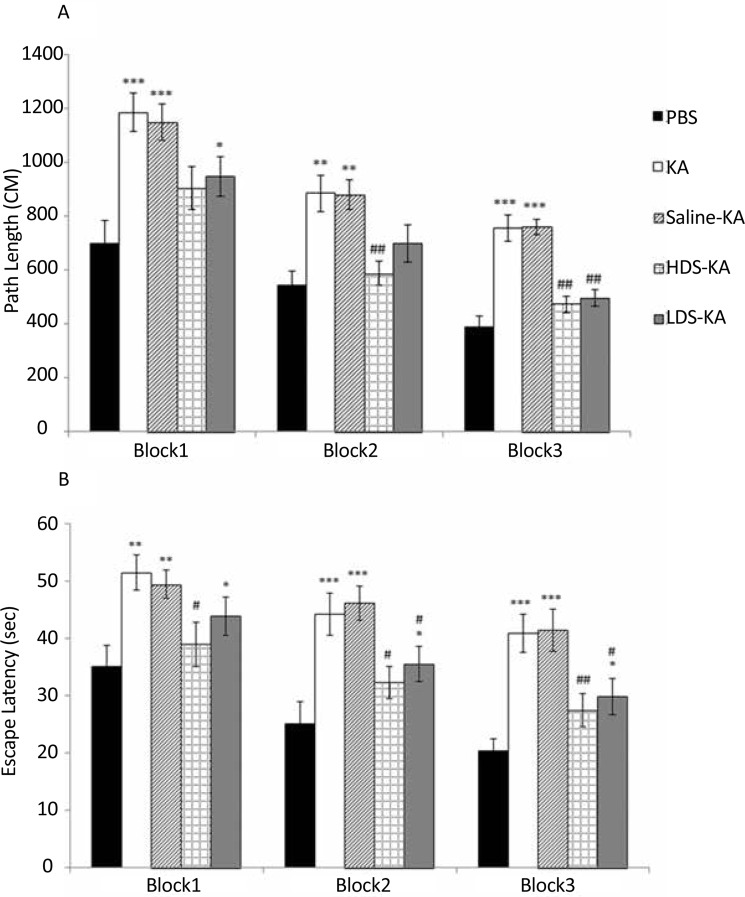
Effects of soy extract on path length (A) and escape latency (B) in finding the hidden platform in the MWM task following KA administration. Seizures in the KA group resulted in significant increase in the traveled distance and escape latency in finding the hidden platform at all three blocks compared with the PBS group. Soy extract at two doses (2 mg/kg in the LDS-KA group and 20 mg/kg in the HDS-KA group) significantly decreased the traveled distance and escape latency to find the hidden platform compared with the KA group. Normal saline in the Saline-KA group did not have significant effects compared with the KA group. *P<0.05, **P<0.01, and ***P<0.001 vs. PBS group; #P<0.05 and ##P<0.01 vs. KA group.

In KA group, the path length (Figure 1A) and escape latency (Figure 1B) significantly enhanced in block 1 (path length: P<0.001, escape latency: P<0.01), block 2 (path length: P<0.01, escape latency: P<0.001), and block 3 (P<0.001) compared to those of the PBS group. Soy extract at the two doses significantly improved such impairment. The path length which traveled by the HDS-KA group in block 2 (P<0.01) and block 3 (P<0.01) and the path length of the LDS-KA group in block 3 (P<0.01) significantly reduced compared to those of KA group (Figure 1A). Furthermore, the escape latency of the HDS-KA group in all three blocks (blocks 1 and 2: P<0.05, block 3: P<0.01) and the escape latency of the LDS-KA group in blocks 2 and 3 (P<0.05) significantly reduced compared to those of KA group (Figure 1B). In the Saline-KA group, the path length and escape latency did not have significant difference compared with the KA group (Figure 1).

#### 3.2.2. Spatial memory

**Figure 2.**
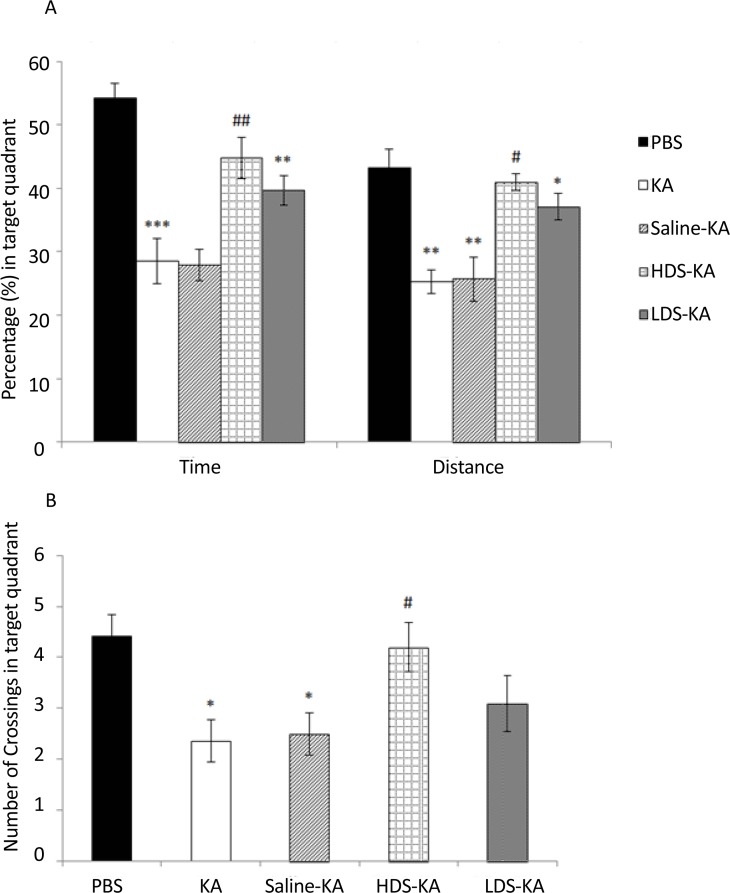
Effects of soy extract on spatial memory impairment following KA administration in the MWM task. Seizures in the KA group resulted in significant decrease in the traveled distance, time spent (A) and the number of crossings (B) in the target quadrant than those of the PBS group. The animals of the HDS-KA group showed significant increase of these three factors in the target quadrant; however, the LDSKA and Saline-KA groups did not show significant effects compared to the KA group. *P<0.05, **P<0.01, and ***P<0.001 vs. PBS group; #P<0.05 and ##P<0.01 vs. KA group.

All factors in the target quadrant, including the time spent (P<0.001), traveled distance (P<0.01) (Figure 2A), and the crossing numbers (P<0.05) (Figure 2B) of the KA group significantly reduced compared to those of the PBS group. In the HDS-KA group, the time (P<0.01), distance (P<0.05) (Figure 2A) and the crossing numbers (P<0.05) (Figure 2B) significantly enhanced compared to those of the KA group. The LDS-KA and Saline-KA groups did not show significant difference with respect to those of the KA group (Figure 2).

#### 3.2.3. Visible test

The speed of swimming and escape latency to discover the visible platform (Table 1) were the same among the groups (P>0.05). Therefore, kainate-induced seizures did not affect motivation or sensory/motor coordination. It is worth noting that since normal saline administration did not have significant effect on the Morris water maze task, we did not conduct the electrophysiology experiment for the Saline-KA group.

**Table 1.** Swimming speed and latency to find the visible platform in the MWM task.

**Groups**	**Swimming Speed (cm/s)**	**Escape Latency (s)**
PBS	20.24±2.4	20.6±2.9
KA	20.68±1.75	25.2±5.3
Saline-KA	20.44±3.08	23.75±6.42
HDS-KA	19.09±1.62	22.75±4.21
LDS-KA	19.14±3.16	20.6±3.57

### 3.3. Field potential recording

#### 3.3.1. Basal excitability of the CA1 area and PPF relationships

**Figure 3.**
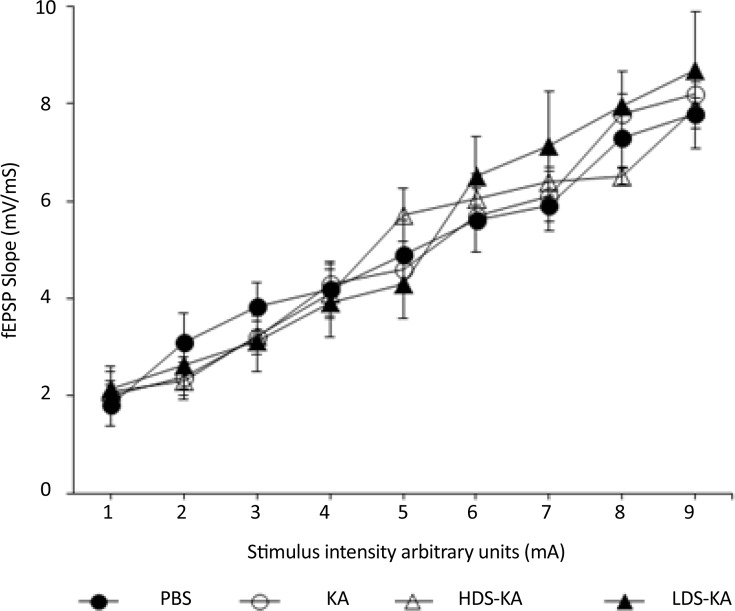
The input-output curve, which shows the effect of seizures on CA1 basal synaptic transmission, was not significantly different between the groups (P>0.05). Arbitrary units are stimulus intensities where 1 is the range of intensities which evoked the minimum response and 9 is the range of intensities which evoked the maximum response.

**Figure 4.**
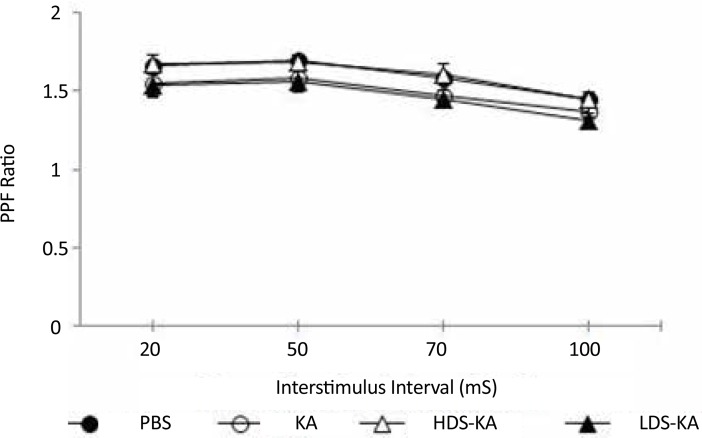
Effects of seizures on CA1 synaptic transmission at various interstimulus intervals (PPF ratios) which was not significantly different between the groups (P>0.05).

The probable effect of seizures on CA1 basal excitability was examined using the input-output curve indicating no significant difference between the groups (P>0.05) (Figure 3). Effect of seizures on CA1 synaptic transmission at various interstimulus intervals (PPF ratios) was also not significantly different among the groups (P>0.05) (Figure 4).

#### 3.3.2. LTP

**Figure 5.**
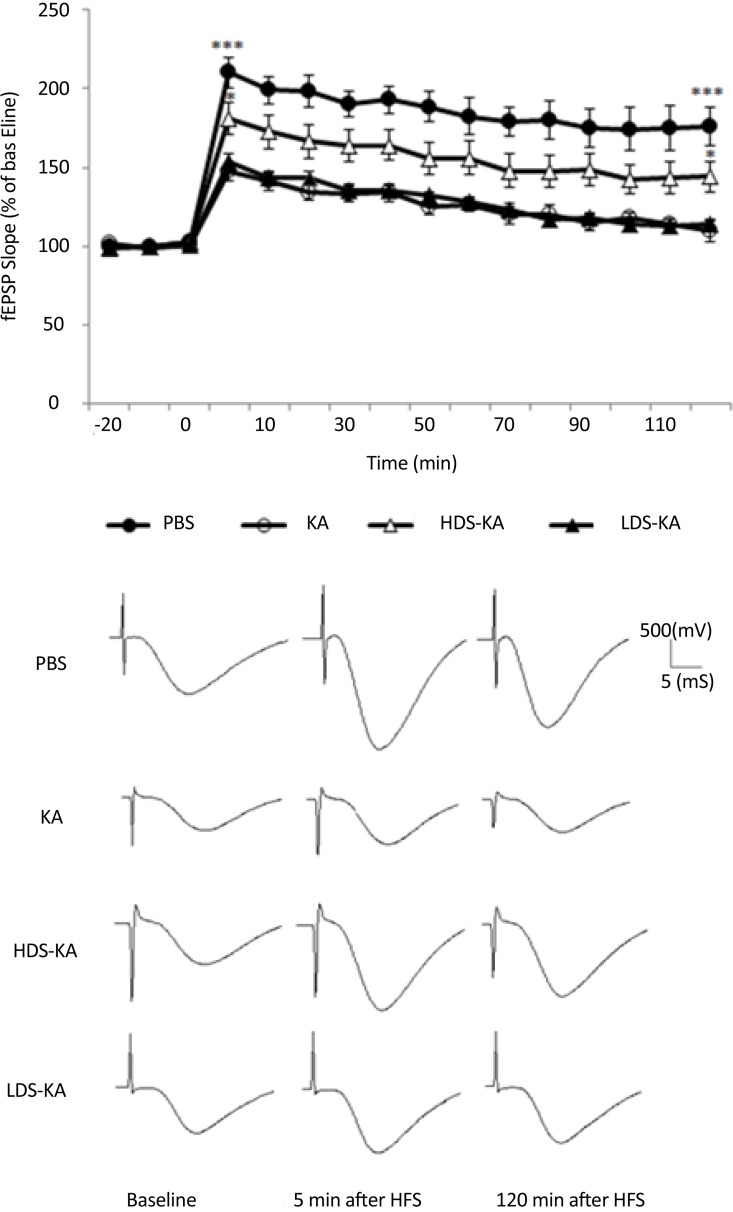
Effects of soy extract on kainate-induced LTP impairment. In the KA group, generalized seizures resulted in significant reduction in LTP induction and maintenance in comparison with the PBS group (P<0.001). Soy extract at the higher dose (in the HDS-KA group) significantly increased LTP induction and maintenance (P<0.05); but, at the lower dose (in the LDS-KA group) it did not have significant effect compared with the KA group (P>0.05). The mean normalized fEPSP slope from every ten sequential traces represents each time point (mean±S.E.M.) in the graph. Calibrations (500 mV/5 ms) were used throughout the experiments. *P<0.05 and ***P<0.001 vs. KA group (at both 5 and 120 min after HFS).

Repeated measurement indicated that LTP significantly decreased at all time points in the KA group relative to the PBS group (P<0.001) (Figure 5). Similar to those of the spatial memory, such overall impairment was improved by soy extract only at the higher dose (20 mg/ kg in the HDS-KA group) compared to those of the KA group (P<0.05). However, the lower dose of the extract (2 mg/kg in the LDS-KA group) did not have significant effect relative to the KA group (P>0.05) (Figure 5). Five minutes after HFS (induction), the slope of the fEPSPs in the KA group showed a significant reduction (P<0.001) (147.2%±5.7% of baseline) than those of the PBS group (210.12%±9.8% of baseline).

The slope of the fEPSPs 5 minutes after HFS showed a significant increase in the HDS-KA group (P<0.05) (180.84%±10.31% of baseline); however, this slope in the LDS-KA group (153.97%±4.75% of baseline) showed no significant difference compared to those of the KA group (P>0.05) (Figure 5). The slope of the fEPSPs 120 minutes after HFS (maintenance) of the PBS (P<0.001) (175.6%±12.2% of baseline) and HDS-KA (P<0.05) (144.18%±9.86% of baseline) groups showed a sustained enhance relative to the KA group (109.8%±6.87% of baseline). However, the slope of the fEPSPs 120 minutes after HFS showed a significant decrease in the LDS-KA group (114.14%±2.18% of baseline) and was not significantly different compared with the KA group (P>0.05) (Figure 5).

## 4. Discussion

We aimed to assess whether or not soy extract containing the phytoestrogen genistein have beneficial effects on kainate-induced cognitive and hippocampal LTP impairment in OVX rats. Soy extract at both higher and lower doses (2 and 20 mg/kg) reduced the destructive effects of the seizure on spatial learning; however, only at the higher dose (20 mg/kg) attenuated spatial memory and hippocampal LTP deficits. The results of this study are consistent with previous studies indicating that epilepsy and seizure induces cognitive and synaptic plasticity impairment (Artinian et al., 2015; Chan et al., 2004; Kotloski et al, 2002; Palizvan et al., 2001; Sgobio et al., 2010) and SPEs and soy extract containing phytoestrogens have beneficial effects on cognitive function (Duffy et al., 2003; Pan, Li, Yeung, & Xu, 2010; Pan, Anthony, Watson, & Clarkson, 2000; Soni et al., 2014). Obviously, seizure induces adverse effects, including neuroinflammation, oxidative stress, and neuronal damage (Ben-Ari, 2001; Patel & Li, 2003; Shapiro et al, 2008).

Seizure can also damage the hippocampus which is crucial for memory formation and, therefore, it may result in memory impairment and LTP deficits in the hippocampus (Azcoitia et al., 2006; Ben-Ari, 2001; Ben-Ari & Represa, 1990; Kotloski et al, 2002; Neves et al, 2008; Palizvan et al., 2001; Sgobio et al., 2010). On the other hand, soy extract containing phytoestrogens have neuroprotective and beneficial properties on cognitive function (Azcoitia et al., 2006; Duffy et al., 2003; Pan et al., 2010; Pan et al., 2000; Soni et al., 2014). Neuroinflammation and oxidative stress can induce cognitive impairment but SPEs have antioxidant properties and protective effects against these abnormalities (Menze, Esmat, Tadros, Abdel-Naim, & Khalifa, 2015; Verdrengh, Jonsson, Holmdahl, & Tarkowski, 2003). Reportedly, similar to that of 17β-estradiol, dietary soy phytoestrogens elevate the expression of Brain-Derived Neurotrophic Factor (BDNF) and synaptic proteins (including spinophilin, synaptophysin, PSD-95 and synapsin 1) in the hippocampus of female OVX rats (Pan et al., 2010). Obviously, BDNF is an important factor for memory formation and LTP induction in the hippocampus (Alonso et al., 2002; Korte, Kang, Bonhoeffer, & Schuman, 1998). Moreover, consumption of soy extract containing phytoestrogens (60 mg total soy isoflavone equivalents/day) results in higher performance of postmenopausal women in the recall of pictures and in sustained-attention and planning tasks (Duffy et al., 2003). Similar to that of 17β-estradiol, administration of soy extract containing SPEs (60 mg/kg/d added to the drinking water) for 10 weeks induces reproduction of the dentate granule cells of old OVX rats (Perez-Martin et al., 2005).

Unlike the destructive effects on LTP, however, seizures have no effects on basal excitability of CA1 neurons or short-term plasticity in this area. Treatment with soy extract also did not affect these factors. Sgobio et al. (2010) also reported similar results. They showed that basal glutamatergic synaptic transmission (I-O curves) and short-term plasticity (PPF ratios) were similar between Bsn mutant epileptic mice and control animals. Treatment with valproic acid also did not affect these factors (Sgobio et al., 2010). However, it has also been shown that seizure can affect basal synaptic transmission and short-term plasticity (Altrock et al., 2003). It seems that the discrepancy between these results may be due to various recording techniques used (Sgobio et al., 2010). Therefore, more studies are needed in this filed to elucidate the effect of seizure on basal synaptic transmission and short-term plasticity.

It has, however, been shown that dietary soy phytoestrogens, decreases febrile seizures threshold in both male and female autistic children (Westmark, 2014). Ebrahimzadeh Bideskan et al. (2011) also reported that soy extract at both 20 and 60 mg/kg doses exacerbate pentylenetetrazol-induced repeated seizures in OVX rats. The present study, however, showed the beneficial effects of soy extract containing the phytoestrogen genistein on spatial memory and LTP deficits following kainate-induced seizure in OVX rats. However, our unpublished data showed that soy extract at both lower and higher doses did not affect seizure activity induced by KA (seizure latency and stages), but significantly decreased hippocampal damage induced by seizure.

Soy extract and SPEs may have dose-dependent effects (Dang & Lowik, 2005). It has been reported that soy extract at higher doses (1 to 20 mg/kg) has neuroprotective effects; however, at a lower dose (0.2 mg/kg) it has no beneficial effects against kainate-induced damage of the hilus of the dentate gyrus in OVX rats (Azcoitia et al., 2006). In our study, soy extract only at the higher dose (20 mg/kg) showed beneficial effects against seizure. Importantly, our and the above-mentioned studies have been conducted under different experimental conditions, such as seizure or epilepsy model which may affect the results. Additionally, beside phytoestrogens, soy extract contains various compounds, including minerals and phenolic compounds (Bolanho & Beléia, 2011; de Lima Toccafondo Vieira, Duarte, Campos, & Nunan Ede, 2008). It is important to note that SPEs, especially genistein, are thought to act via interaction with Estrogen Receptors (ERs) and therefore mimic the actions of 17β-estradiol (Molteni et al., 1995). Therefore, the beneficial effects of SPEs on seizure might be mediated via ERs.

Overall, the results suggest that soy extract containing the phytoestrogen genistein has beneficial effects against seizure-induced cognitive impairment. It seems that such beneficial neurobehavioral effects of soy extract are accompanied by a beneficial effect on hippocampal synaptic plasticity. However, further studies on the current topic are required.
